# Strengthening Systems: Advancing Integrated Behavioral Health for Older Adults

**DOI:** 10.1093/geroni/igaf122.650

**Published:** 2025-12-31

**Authors:** Allison Gibson, Kelly O’Malley

## Abstract

Implementing integrated behavioral health in aging services presents several challenges, including organizational silos, cultural differences between sectors, stigma, and a lack of trained staff who are equipped to address both aging and behavioral health needs. Workforce shortages and the complexity of older adults’ health needs further complicate integration efforts. This symposium will highlight a series of strategies to overcome these challenges, including systems organizational change, improving training and care coordination, enhancing students’ training in geriatric competencies and workforce development, and increasing access to appropriate resources. The first session discusses collaboration processes with aging-focused and behavioral health providers to foster cross-sector integration. The discussion highlights the importance of system change in making aging services and behavioral health more comprehensive and responsive to older adults. Our second presentation explores utilizing the 4Ms-Behavioral Health Framework to support integration efforts by examining data on behavioral health and substance use among older adults in the U.S. and Australia, emphasizing how training in different settings can lead to meaningful knowledge and behavioral change among interprofessional teams. Our third presentation prepares students to apply geriatric care competencies through the Mobile Health and Wellness Program (MHWP). Presenters will discuss how this initiative enhances workforce development, preparing future providers to integrate aging and behavioral health care effectively. Our final presentation will discuss the role of the Comprehensive Healthcare Integration (CHI) Framework, which enhances patient-centered care by embedding behavioral health consultants into a team-based model that improves health outcomes and care coordination of older clients.

